# Association Between Tumor-Infiltrating Immune Cells and Early Recurrence in Ampulla of Vater Carcinoma

**DOI:** 10.7759/cureus.100278

**Published:** 2025-12-28

**Authors:** Kyohei Yoshino, Tomohiko Adachi, Takayuki Tanaka, Hajime Matsushima, Hajime Imamura, Takanobu Hara, Akihiko Soyama, Kengo Kanetaka, Susumu Eguchi

**Affiliations:** 1 Department of Surgery, Nagasaki University Graduate School of Biomedical Sciences, Nagasaki, JPN; 2 Department of Digestive and General Surgery, Shimane University Faculty of Medicine, Shimane, JPN

**Keywords:** ampulla of vater carcinoma, early recurrence, gastrointestinal surgery, immunohistochemistry, pd-1, pd-l1, prognostic factors, tumor-infiltrating immune cells

## Abstract

Introduction

Ampulla of Vater carcinoma (AVC) is a rare gastrointestinal malignancy with limited evidence from large-scale clinical studies. Although curative resection remains the standard treatment, early recurrence significantly worsens prognosis. Increasing attention has been paid to the tumor immune microenvironment, particularly immune checkpoint molecules, which influence tumor behavior. This study aimed to identify clinicopathological and immunological factors associated with early recurrence in AVC.

Methods

We retrospectively analyzed 42 patients who underwent curative resection for AVC at Nagasaki University Hospital between October 2005 and March 2022. Early recurrence was defined as tumor relapse within one year after surgery. Immunohistochemical staining was performed for tumor-infiltrating immune cells, including CD4, CD8, PD-1, PD-L1, Foxp3, and TIM3. Statistical analyses included Fisher’s exact test, Mann-Whitney U test, and Kaplan-Meier survival analysis.

Results

Seven patients (16.7%) experienced early recurrence. Compared with the non-early recurrence group, the early-recurrence group had significantly higher preoperative CA19-9 levels (p = 0.009) and higher incidences of vascular invasion (p = 0.002), perineural invasion (p = 0.002), lymph node metastasis, and advanced Union for International Cancer Control (UICC) stage (≥ IIB; both p < 0.05). Immunohistochemically, the early-recurrence group showed significantly increased expression of CD4⁺ T cells, PD-1, and PD-L1 (p < 0.001, 0.004, and < 0.001, respectively), whereas CD8, Foxp3, and TIM3 expression did not differ significantly. Kaplan-Meier analysis demonstrated significantly poorer overall survival in the early-recurrence group (p < 0.001).

Conclusion

Early recurrence of AVC is strongly associated with aggressive pathological features and distinct immune profiles, including increased expression of CD4⁺ T cells, PD-1, and PD-L1. These findings suggest a potential association between the tumor immune microenvironment and early recurrence in AVC. However, the present results should be interpreted as exploratory and hypothesis-generating. Further large-scale studies are warranted to clarify the biological significance of these immune features and their potential therapeutic implications.

## Introduction

Ampulla of Vater carcinoma (AVC) is a rare malignancy, accounting for approximately 6% of peripancreatic cancers and only 0.2% of all gastrointestinal cancers [1,2]. Its incidence has shown a modest annual increase of 0.9% [3]. However, due to its rarity and the lack of large-scale studies, the epidemiological characteristics of AVC remain poorly understood [4,5]. Pancreaticoduodenectomy (PD) is considered the standard curative treatment for AVC, yet recurrence rates remain high, with a five-year survival rate of approximately 45% post-resection [6]. This poor prognosis stems from a lack of established, effective systemic therapies and the challenges posed by the disease's rarity. Furthermore, the absence of large-scale prospective studies further complicates efforts to improve treatment strategies. The anatomical complexity of the duodenal papilla‒comprising the pancreatic duct, distal bile duct, and duodenal mucosa‒adds to the difficulty of developing standardized treatment approaches. Histopathologically, AVC has been classified into biliopancreatic (PB) and intestinal (IT) subtypes to assess their prognostic implications. This classification was originally proposed by Kimura et al. in 1994 [7], and subsequent studies have consistently shown that PB-type tumors are associated with poorer outcomes compared to IT-type tumors [8-11]. However, conflicting reports suggest no significant oncologic differences between the two subtypes in some studies [12-15]. In pathology, immunohistochemistry is widely employed for diagnostic purposes [16]. Given the rarity of AVC, the immunological characteristics of this disease remain poorly understood, especially the involvement of tumor-infiltrating immune cells in recurrence, making it clinically important to clarify these immunological features. However, how the tumor immune microenvironment contributes to early recurrence after curative resection in AVC has not been systematically investigated.

Advances in tumor immunology have highlighted the importance of immune checkpoint pathways, particularly PD-1 and PD-L1, in shaping the tumor microenvironment (TME) [17]. These discoveries have led to the widespread adoption of immune checkpoint inhibitors (ICIs) as treatment modalities for various cancers [18-20]. The role of tumor immunity has thus become a pivotal factor in cancer control, regardless of cancer type [17]. TIM-3, expressed on CD4⁺ and CD8⁺ T cells, contributes to T cell exhaustion through interactions with tumor cells, making it a marker of lymphocyte exhaustion similar to PD-1 [21]. Conversely, Foxp3, predominantly expressed in Tregs, promotes an immunosuppressive environment [22]. A detailed understanding of these immunological pathways is essential for developing effective tumor management strategies across cancer types. Given the significant impact of early recurrence on patient prognosis [23], this study aimed to identify clinicopathological and immunological factors associated with early recurrence in AVC. Specifically, we performed immunohistochemical analyses of tumor-infiltrating immune cells, including CD4, CD8, PD-1, PD-L1, Foxp3, and TIM-3, comparing their expression levels between patients with and without early recurrence.

## Materials and methods

Study population

This study was approved by the Institutional Review Board of Nagasaki University Hospital (Approval No. 19102143) and conducted in accordance with the Declaration of Helsinki. The requirement for written informed consent was waived by the Institutional Review Board due to the retrospective nature of the study. A consecutive cohort of patients with AVC who underwent surgical resection for primary tumors with curative intent at Nagasaki University Graduate School between October 2005 and March 2022 was analyzed. A total of 42 patients with carcinoma of the AVC were included in the study. Other periampullary cancers, including distal common bile duct cancer, pancreatic head cancer, and duodenal cancer, were excluded. Among the 42 cases, 23 patients underwent open surgery, 17 underwent laparoscopic surgery (with two conversions to open surgery), and two underwent robotic surgery. No patient received neoadjuvant therapy. Adjuvant chemotherapy was administered in 16 of 42 patients (38%), predominantly with S-1 or gemcitabine-based regimens. Among them, adjuvant chemotherapy was given in 5 of 7 patients (71%) in the early recurrence group and 11 of 35 patients (31%) in the non-early recurrence group. Clinicopathological and survival outcome data were obtained from the electronic medical records at Nagasaki University Graduate School. Tumors were staged using the seventh Union for International Cancer Control (UICC) staging system [24]. Early recurrence was defined as tumor recurrence within one year after the initial surgery, based on previously published studies [23]. Postoperative surveillance was performed according to our standard institutional follow-up protocol, which generally includes regular outpatient visits with laboratory testing and cross-sectional imaging at predefined intervals, with additional evaluations as clinically indicated.

Immunohistochemistry (IHC) analysis

The selected immunohistochemical markers (CD4, CD8, PD-1, PD-L1, Foxp3, and TIM-3) were chosen to comprehensively evaluate key components of the tumor immune microenvironment, including T-cell subsets, immune checkpoint pathways, regulatory T cells, and exhaustion-related markers. Tissue slides (5μm thick) were prepared from formalin-fixed, paraffin-embedded (FFPE) samples. After deparaffinization, the slides were rehydrated through graded ethanol solutions. Antigen retrieval was performed by boiling the slides in ethylenediaminetetraacetic acid (EDTA) buffer (1 mM, pH 9.0) using a microwave oven. Endogenous peroxidase activity was blocked using Dako REAL Peroxidase-Blocking Solution (S2023, Agilent, Santa Clara, CA), and non-specific binding was prevented by incubating the slides with a ready-to-use protein block (serum-free, X0909, Agilent, Santa Clara, CA). The slides were then incubated overnight with primary antibodies: rabbit anti-human PD-1 (Abcam, ab52587, 1:100), rabbit anti-human TIM3 (Abcam, ab241332, 1:100), mouse anti-human CD8 (Leica, NCL-L-CD8-4B11, 1:50), and rabbit anti-human CD4 (Roche, 790-4460, 1:30, Switzerland). Next, the slides were treated with Dako REAL EnVision/HRP, Rabbit/Mouse (K5007, Agilent, Santa Clara, CA), followed by incubation with diaminobenzidine (DAB) solution (Dako REAL DAB Chromogen Bottle C, diluted 1:100 in Dako REAL Substrate Buffer Bottle B, K5007, Agilent, Santa Clara, CA) to visualize immunoreactivity. After counterstaining with hematoxylin, the slides were dehydrated and mounted. Immunostaining for PD-L1 and Foxp3 was outsourced to Morphotechnology Inc. (Tokyo, Japan). PD-L1 expression was evaluated on tumor-infiltrating immune cells rather than on tumor cells, in accordance with previous studies investigating the tumor immune microenvironment. For analysis, hot spots were defined as areas showing the highest density of immunoreactive cells within the tumor, identified at ×400 magnification. Three hot spots from immunoreactive slides were selected, and the area ratio was quantified using image analysis software (WinROOF®, Mitani Corp., Tokyo, Japan). The area ratio was defined as the percentage of immunoreactive area within the selected region of interest, rather than the proportion of positive cells among total tumor-infiltrating lymphocytes. The average value from these measurements was used for further analysis.

Statistical analysis

Statistical analyses were performed using EZR (Easy R), version 1.55 (Saitama Medical Center, Jichi Medical University, Saitama, Japan) software. Fisher's exact test was used to analyze categorical variables, while the Mann-Whitney U test was used for continuous variables. A p-value of less than 0.05 was considered statistically significant. A log-rank test was used to compare differences between Kaplan-Meier curves. Because this was a retrospective study of a rare malignancy, a formal a priori sample size calculation was not performed; instead, all eligible consecutive cases during the study period were included.

## Results

Clinicopathological characteristics

A total of 42 patients who underwent curative resection for ampulla of Vater carcinoma were analyzed. Patients were divided into an early-recurrence group (n = 7) and a non-early-recurrence group (n = 35) based on recurrence within 12 months; baseline characteristics are summarized in Table 1.

**Table 1:** Comparison of clinicopathological characteristics between the early-recurrence and non-early-recurrence groups. Baseline clinicopathological characteristics of patients stratified by early-recurrence status after curative resection for ampulla of Vater carcinoma. Continuous variables are presented as median (interquartile range), and categorical variables as number (percentage). Statistical comparisons were performed using the Mann-Whitney U test or χ²/Fisher’s exact test as appropriate. p < 0.05 was considered statistically significant. CA19-9, carbohydrate antigen 19-9; CEA, carcinoembryonic antigen; UICC, Union for International Cancer Control.

Variables		Early recurrence (-) (n=35)	Early recurrence (+) (n=7)	p
Age, Median (Range)		71 (43-86)	78 (59-82)	0.362
Sex	Male/female	13/22	2/5	1.000
Preoperative serum CA19-9 (U/ml)	<37 (n=21)	21	0	0.009
	≧37 (n=21)	14	7	
Preoperative serum CEA (ng/ml)	<5 (n=36)	30	6	1.000
	≧5 (n=6)	5	1	
Tumor size (mm)	<20 (n=20)	16	4	0.691
	≧20 (n=22)	19	3	
Vascular invasion	No (n=23)	22	1	0.002
	Yes (n=19)	13	6	
Lymphatic invasion	No (n=19)	18	1	0.099
	Yes (n=22)	16	6	
Perineural invasion	No (n=27)	26	1	0.002
	Yes (n=12)	1	6	
Duodenum invasion (T2)	No (n=18)	17	1	0.208
	Yes (n=24)	18	6	
Pancreatic invasion (T3)	No (n=28)	26	2	0.031
	Yes (n=14)	9	5	
Peripancreatic head lymph node metastasis	No (n=27)	25	2	0.077
	Yes (n=15)	10	5	
Lymph node metastasis	0 (n=24)	23	1	0.031
	1 (n=18)	12	6	
UICC stage	<2B (n=24)	23	1	0.031
	≧2B (n=18)	12	6	

Preoperative CA19-9 levels were significantly higher in the early-recurrence group (p = 0.009). Pathological adverse features, including vascular invasion and perineural invasion, were also more frequently observed in this group (both p = 0.002). In contrast, lymphatic invasion did not differ significantly between the two groups (p = 0.099). In addition, lymph node metastasis and advanced UICC stage (≥ IIB) were more common among patients with early recurrence (both p < 0.05). No significant differences were observed in age, sex, tumor size, or serum CEA levels between the two groups. These findings indicate that aggressive pathological features are strongly associated with early recurrence in AVC.

Immunohistochemical findings

**Figure 1:**
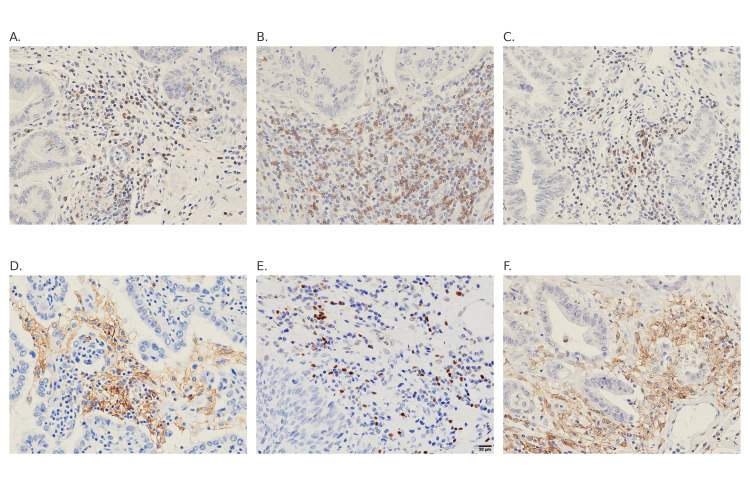
Representative immunohistochemical staining for tumor-infiltrating immune markers. (A) CD4, (B) CD8, (C) PD-1, (D) PD-L1, (E) Foxp3, and (F) TIM3 staining in ampulla of Vater carcinoma tissues. Staining patterns and intensities were compared between the early-recurrence and non-early-recurrence groups. A representative panel includes a scale bar indicating 100 μm.

Representative immunohistochemical staining of tumor-infiltrating immune cells (CD4, CD8, PD-1, PD-L1, Foxp3, and TIM3) is shown in Figure 1, and quantitative analyses are presented in Table 2.

**Table 2:** Comparison of tumor-infiltrating immune cell expression between the early-recurrence and non-early-recurrence groups. Expression levels of tumor-infiltrating immune markers (PD-L1, PD-1, Tim-3, CD4, CD8, and FoxP3) were evaluated by immunohistochemical staining and quantified as the percentage of immunoreactive area within representative high-power fields using image analysis software (WinROOF®, Mitani Corporation, Fukui, Japan), rather than as the proportion of positive cells among total tumor-infiltrating lymphocytes. Data are presented as median (interquartile range). Statistical comparisons between the early-recurrence and non-early-recurrence groups were performed using the Mann-Whitney U test, and p < 0.05 was considered statistically significant. PD-1, programmed cell death protein 1; PD-L1, programmed death-ligand 1; Tim-3, T-cell immunoglobulin and mucin-domain-containing-3.

Variables	Univariate analysis		
	Early recurrence (-) (n=35)	Early recurrence (+) (n=7)	p
CD4	1.527	3.281	<0.001
CD8	1.628	1.006	0.140
PD-1	0.578	2.305	0.004
PD-L1	0.817	3.286	<0.001
Foxp3	0.449	0.772	0.353
TIM3	1.160	2.373	0.370

The early-recurrence group exhibited significantly higher expression of CD4⁺ T cells, PD-1, and PD-L1 (p < 0.001, 0.004, and < 0.001, respectively) compared with the non-early-recurrence group, whereas no significant intergroup differences were observed for CD8, Foxp3, or TIM3. These findings suggest that tumors in the early-recurrence group exhibit an immunosuppressive microenvironment characterized by enhanced immune checkpoint activation (PD-1/PD-L1 axis) and increased CD4⁺ T cell infiltration.

Survival analysis

**Figure 2:**
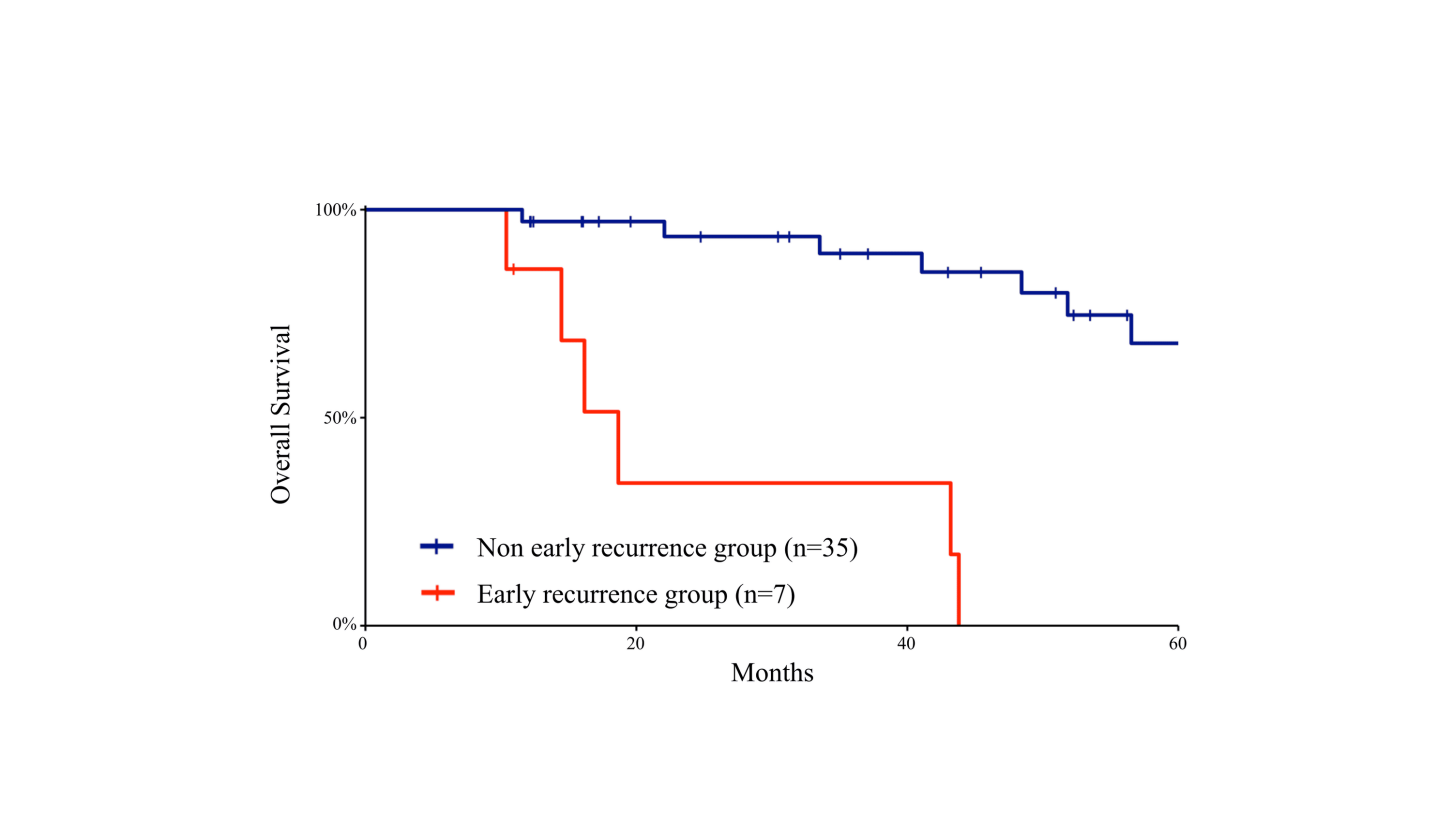
Kaplan-Meier curves for overall survival analysis comparing the early-recurrence and non-early-recurrence groups after curative resection of ampulla of Vater carcinoma. Kaplan-Meier curves for overall survival analysis comparing the early-recurrence and non-early-recurrence groups after curative resection of ampulla of Vater carcinoma. Overall survival was estimated using the Kaplan-Meier method, and differences between the two groups were analyzed using the log-rank test. The early-recurrence group demonstrated significantly shorter overall survival compared with the non-early-recurrence group.

Kaplan-Meier curves for overall survival are shown in Figure 2.

Patients in the early-recurrence group demonstrated significantly shorter overall survival compared with those in the non-early-recurrence group (p < 0.001, log-rank test).

Quantification of immune cells using image analysis

**Figure 3:**
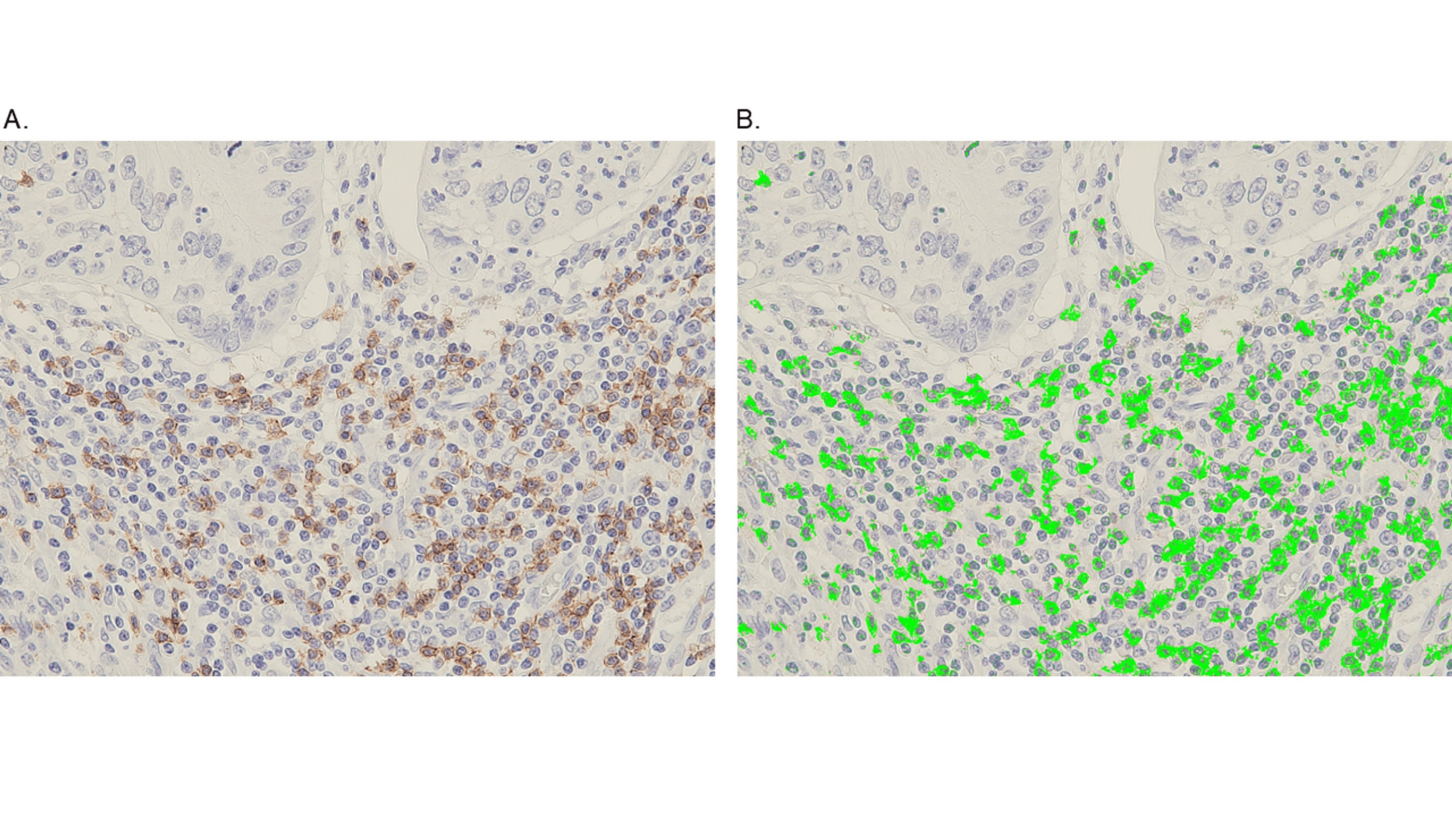
Representative image illustrating the quantification of tumor-infiltrating immune cells using WinROOF®. Representative image showing the quantification of tumor-infiltrating immune cells by digital image analysis using WinROOF® software (Mitani Corporation, Fukui, Japan). Positively stained immune cells were automatically identified and counted within selected regions of interest under high-power magnification. The quantified data were used for statistical comparison of immune marker expression between the early-recurrence and non-early-recurrence groups.

Figure 3 shows an example of immune cell quantification using the digital image analysis software WinROOF®.

Positively stained cells within predefined regions of interest under high-power magnification were automatically identified, and the resulting area ratios were used for statistical comparisons between the two groups.

## Discussion

In this study, we investigated the clinicopathological and immunological factors associated with early recurrence in patients with AVC and identified characteristics linked to this recurrence. Among the clinicopathological factors, elevated preoperative CA19-9 levels, vascular invasion, perineural invasion, tumor depth classified as T3 or greater, lymph node metastasis, and UICC stage 2B or higher were significantly correlated with early recurrence. In contrast, lymphatic invasion did not differ significantly between the two groups in our cohort, which should be considered when interpreting the clinicopathological correlates of early recurrence, suggesting that vascular and lymphatic invasion may have distinct biological and prognostic implications in AVC. These findings are consistent with previous studies, which have shown that the pancreatobiliary (PB) type of AVC is frequently associated with advanced tumor stage, lymph node involvement, and perineural invasion [25]. However, the limited sample size of the early recurrence group precluded histological classification into PB and intestinal (IC) subtypes based on immunostaining, leaving the prevalence of PB-type tumors in this group uncertain. Further studies with larger sample sizes are needed to clarify this relationship.

From an immunological perspective, PD-L1 and PD-1 expression levels were significantly higher in the early recurrence group, although CD8+ T cell infiltration did not differ significantly between groups. The PD-1/PD-L1 pathway is a critical immune checkpoint exploited by tumors to evade immune surveillance, leading to T cell exhaustion and diminished anti-tumor immunity. Elevated PD-L1 expression has been associated with poor prognosis in several cancers [26]. ICIs targeting this pathway have shown efficacy in various malignancies, such as gastric and esophageal cancers [27]. However, their role in AVC, particularly concerning early recurrence, remains underexplored. Although CD8+ T cell expression did not significantly differ between groups, the elevated PD-L1 expression suggests an immunosuppressive TME in the early recurrence group. This scenario may reflect a "cold tumor" phenotype characterized by immune evasion mechanisms and limited cytotoxic T cell activity [28]. However, the absence of a significant decrease in CD8+ T cell expression suggests that the immunological landscape of AVC may not entirely align with the classical "cold tumor" definition, potentially involving other immunosuppressive mechanisms or dysfunctional T cell activity. The significantly higher expression of CD4+ T cells in the early recurrence group is also noteworthy. CD4+ T cells include various subsets, such as Tregs and helper T cells, which can either suppress or promote tumor progression. In this study, Foxp3 expression, a marker of Tregs, was not significantly elevated in the early recurrence group. This finding implies that the increase in CD4+ T cells may involve other subsets, such as Th17 or Th2 cells. Th17 cells secrete cytokines like IL-17A and IL-22, which are known to enhance tumor proliferation, angiogenesis, and metastasis [29]. Similarly, Th2 cells produce cytokines such as IL-4 and IL-13, contributing to an immunosuppressive TME [30]. These dynamics highlight the complex roles of CD4+ T cell subsets in the TME and their potential impact on tumor progression. Recent clinical trials have demonstrated that ICIs targeting the PD-1/PD-L1 pathway can improve outcomes in various cancers [31]. However, the effectiveness of these therapies may be limited by an immunosuppressive TME [26]. In patients with AVC with early recurrence and high PD-L1 expression, the presence of immunosuppressive CD4+ T cell subsets and other inhibitory mechanisms may reduce the efficacy of ICIs [28]. Given the immunosuppressive nature of AVC’s tumor microenvironment, a combination approach that not only blocks immune checkpoint pathways but also counteracts the influence of Th2- and Th17-driven immune responses could be crucial in improving therapeutic efficacy. In particular, interventions that address T cell dysfunction within the TME, rather than merely increasing T cell infiltration, may be necessary to enhance anti-tumor immunity.

Future research should explore the potential of integrating ICIs with immunomodulatory therapies that restore effective anti-tumor immune responses, ultimately refining treatment strategies for AVC. Several pathological factors, including vascular invasion, perineural invasion, lymph node metastasis, and tumor stage, are well established as being associated with recurrence after curative resection of ampulla of Vater carcinoma. Therefore, the interpretation of tumor-infiltrating immune cells should be considered in the context of these adverse clinicopathological features. Because of the rarity of this disease and the limited sample size of the present cohort, particularly the small number of patients with early recurrence, multivariate analysis to adjust for these potential confounding factors was not feasible. Accordingly, this study should be regarded as an exploratory, hypothesis-generating analysis rather than a definitive assessment of independent prognostic factors. Nevertheless, our findings suggest a potential association between the tumor immune microenvironment and early recurrence in ampulla of Vater carcinoma, which may provide a rationale for future large-scale, multicenter studies designed to clarify the independent prognostic and biological roles of tumor-infiltrating immune cells.

Limitations

This study has several limitations. First, it was a single-center retrospective analysis with a relatively small sample size, which may limit the generalizability of the findings. In addition, although the study period was relatively long, the immunohistochemical staining procedures and evaluation methods were performed using consistent protocols throughout the study period. Second, immunohistochemical evaluation of tumor-infiltrating lymphocytes was semi-quantitative and may have been influenced by interobserver variability. Third, although we examined multiple immune markers, other factors influencing the tumor immune microenvironment were not assessed. Further multi-institutional studies with larger cohorts and standardized immunologic analyses are warranted to validate these findings.

## Conclusions

In summary, this study demonstrated that CD4⁺ T cells, PD-1, and PD-L1 were more highly expressed in patients with early recurrence of ampulla of Vater carcinoma. These findings suggest an association between the tumor immune microenvironment and early recurrence in AVC, rather than establishing a definitive immunosuppressive phenotype. Although immune checkpoint pathways may be involved in this process, the present results should be interpreted as exploratory and hypothesis-generating. Further large-scale studies are warranted to validate these observations and to clarify the potential biological and clinical implications of immune checkpoint-related pathways in AVC.
